# Estimation of Heart Rate Using Regression Models and Artificial Neural Network in Middle-Aged Adults

**DOI:** 10.3389/fphys.2021.742754

**Published:** 2021-09-30

**Authors:** Kuan Tao, Jiahao Li, Jiajin Li, Wei Shan, Huiping Yan, Yifan Lu

**Affiliations:** ^1^School of Sports Engineering, Beijing Sport University, Beijing, China; ^2^School of Sport Medicine and Physical Therapy, Beijing Sport University, Beijing, China; ^3^China Institute of Sport and Health Science, Beijing Sport University, Beijing, China; ^4^Key Laboratory of Sports and Physical Fitness of the Ministry of Education, Beijing Sport University, Beijing, China

**Keywords:** regression model, correlation analysis, maximal heart rate, artificial neural network, heart rate estimation

## Abstract

**Purpose**: Heart rate is the most commonly used indicator in clinical medicine to assess the functionality of the cardiovascular system. Most studies have focused on age-based equations to estimate the maximal heart rate, neglecting multiple factors that affect the accuracy of the prediction.

**Methods**: We studied 121 middle-aged adults at an average age of 57.2years with an average body mass index (BMI) of 25.9. The participants performed on a power bike with a starting wattage of 0W that was increased by 25W every 3min until the experiment terminated. Ambulatory blood pressure and electrocardiography were monitored through gas metabolic analyzers for safety concerns. Six descriptive characteristics of participants were observed, which were further analyzed using a multivariate regression model and an artificial neural network (ANN).

**Results**: The input variables for the multivariate regression model and ANN were selected by correlation for the reduction of dimension. The accuracy of estimation by multivariate regression model and ANN was 9.74 and 9.42%, respectively, which outperformed the traditional age-based model (with an accuracy of 10.31%).

**Conclusion**: This study provides comprehensive approaches to estimate the maximal heart rate using multiple indicators, revealing that both the multivariate regression model and ANN incorporated with age, resting heart rate (RHR), and second-order heart rate (SOHR) are more accurate than univariate models.

## Introduction

Heart rate is the most commonly used indicator in clinical medicine to assess the functionality of the cardiovascular system (Fox et al., 2007). Biological systems exhibit metrics described by heart rate to measure complex physical and psychological challenges (Shaffer and Ginsberg, 2017). Heart rate variability is the fluctuation in the time intervals between adjacent heartbeats, which underlies human attention and emotional states (McCraty and Shaffer, 2015). Maximal heart rate serves as a surrogate marker of peak performance in the exercise test (Gelbart et al., 2017), while the target heart rate, defined as 55–90% of the maximal heart rate (Swain et al., 1994), is reported to represent the prescribed exercise intensities by the American College of Sports Medicine (ACSM; da Silva et al., 2020). Heart rate estimation of middle-aged groups is especially important as they are at risk for cardiovascular and other chronic diseases.

In the most common predictive model, univariate linear regression is generally applied to estimate the maximal heart rate using traditional age-based equations proposed by Fox et al. (1971) and Tanaka et al. (2001). Although these equations have been extensively examined in specific categories, such as healthy and sedentary adults (Nes et al., 2013; Sarzynski et al., 2013), their practical applications remain to be discussed, as previous studies found that both formulas overestimated the maximal heart rate for female recreational marathon runners (Nikolaidis et al., 2018) and Brazilian jiu-jitsu athletes (Branco et al., 2020). Multivariate statistical models are also constructive when the target variable is dependent on input variables. For example, hemoglobin is shown to be associated with age, gender, body mass index (BMI), and potential interactions between these covariates. Hence, the multivariate approach detects these interrelated hematological indices and provides more informative results than univariate linear regression (McArtor et al., 2017). Advances in heart rate estimation utilize robust machine-learning techniques. For instance, K-means clustering is used to separate noisy and non-noisy data collected from bio-monitoring devices, and then a random forest model is applied to predict heart rate (Bashar et al., 2019). Next, a support vector machine (SVM) with a radial basis function (RBF) kernel is proposed for remote video-based heart rate estimation (Osman et al., 2015). In addition, a sophisticated artificial neural network (ANN) algorithm, which is based on the gradient descent of the loss function, provides a nonlinear relation between the target variable and multiple input variables by minimizing the deficit between the true value and the estimated value of the heart rate (Zheng et al., 2017; Norouzian et al., 2021; Wei and Yang, 2021).

Despite numerous studies on the estimation of heart rate *via* multiple factors, to the best of our knowledge, no study has incorporated first-and second-order heart rates (SOHRs), even though these low-level load heart rates are critical to measure the maximal exercise capacity of participants. This study aims to establish a comprehensive multivariate model of the selected variables, and an ANN model to predict the heart rate considering six factors (age, BMI, resting heart rate (RHR), and first-and SOHR) for middle-aged groups.

## Materials and Methods

### Participants

A total of 121 middle-aged adults (age ranging from 41 to 71years) in Beijing, with an average age of 57.2years and an average BMI of 25.9 were enrolled. Participants with a history of heart disease, asthma, peripheral vascular disease, cerebrovascular disease, diabetes mellitus, kidney disease, or recent lower extremity injury were excluded from the study. All recruited subjects visited the laboratory for screening and signed an informed consent form 1week prior to the beginning of the study. All participants were instructed to avoid performing heavy and prolonged exercise and to rest on the day before and the day of the test. The test started 0.5h after the participants ate. Alcoholic drinks were prohibited on the day of the test, and coffee and tea consumption was ceased at least 1h before the test. This research was approved by the ethics committee from Beijing Sport University and complied with the Declaration of Helsinki. All participants have provided informed consent at the time of enrollment.

### Graded Exercise Test

Participants exercised on a power bike until symptoms were limited and the heart rate at the cease moment, which we defined as the “maximum heart rate” (HR_max_). As per the absolute indications for terminating a symptom-limited maximal exercise test recommended by ACSM, the term of the subject’s request to stop was adopted during the experiment. The starting wattage of each test was 0W. After test initiation, the wattage was increased by 25W every 3min until the subject failed to maintain it. The experiment was terminated based on the ACSM criteria: (1) heart rate 85% or more of the expected maximum heart rate, (2) respiratory quotient greater than 1.10, (3) oxygen uptake plateaued or decreased with increasing exercise intensity, and (4) exertion of maximum force and inability of the participant to maintain the prescribed load. Testing was terminated if at least three of the aforementioned criteria were simultaneously reached. The participants wore gas metabolic analyzers and were monitored for ambulatory blood pressure and ambulatory electrocardiography during the experiment. In addition, the reactions and symptoms of each subject were observed and recorded during exercise to ensure safety.

Table 1 displays the descriptive characteristics of participants, including three indices (age, BMI, and RHR) reflecting basic information before exercise, and values on two indices (first-and second-order heart rate) mirroring whether participants had exercise habits. The maximum heart rate, defined under the settings of our experiments, was also collected. Information on male and female participants was listed separately; however, no significant differences were noted between the maximum heart rates of men and women (*p*>0.05).

**Table 1:** Subject Information.

Indicators	Total (*N*=121)	Male (*N*=62)	Female (*N*=58)
Age (years)	57.2±6.4	57.4±7.0	57.0±5.8
BMI (kg.m^-2^)	25.9±2.7	26.0±2.7	25.8±2.7
RHR (bpm)	77.5±11.5	77.7±12.1	77.3±11.0
FOHR (bpm)	96.9±12.1	93.5±12.2	100.6±10.9
SOHR (bpm)	113.0±15.6	105.2±13.4	121.0±13.5
HRmax (bpm)	151.4±19.6	151.4±19.7	151.4±19.7

RHR, resting heart rate; BMI, body mass index; FOHR, first-order heart rate; and SOHR, second-order heart rate.

### Univariate and Multivariate Model

The collected data were processed using R (version 3.6) and Python (version 3.7). Independent sample *t*-tests for maximum heart rate between men and women were performed in R language, along with the correlation between RHR, first-order heart rate (FOHR), SOHR, BMI, age, gender, and maximum heart rate using the PerformanceAnalytics package. The relationship between age and maximum heart rate was calculated in this experiment using univariate linear regression, whereas a multivariate regression model was constructed using the MASS package to filter variables. The variables are selected from a set containing six labels and are introduced into the model one by one to ensure that the addition of each variable results in a higher accuracy of heart rate estimation. Hence, an optimal linear model is obtained.

### Artificial Neural Network Model

The data were randomly divided into two subsets. The first subset was the training set (*N*=96) with the *K*-fold cross-validation method (*K*=5), and the second subset was the testing set (*N*=24), which was used to evaluate the robustness of the proposed model. In this study, the input layer was filled with data labeling with age, gender, BMI, RHR, FOHR, and SOHR, while the output layer indicated the predicted heart rate. To enhance the accuracy of the estimation, the total network comprised four layers, including two hidden layers with 128 neurons per layer, which were fully connected to the input and output layers. We defined the loss function as the bias between the predicted and true values under the mean square error (MSE). In addition, the hyperparameter learning rate and epoch were set as 0.023 and 5,000, respectively, and the activation function was chosen as ReLu. A backpropagation algorithm equipped with a stochastic gradient descent method was adopted to minimize the loss and efficiently update 18,069 parameters of the ANN.

## Results

**Figure 1:**
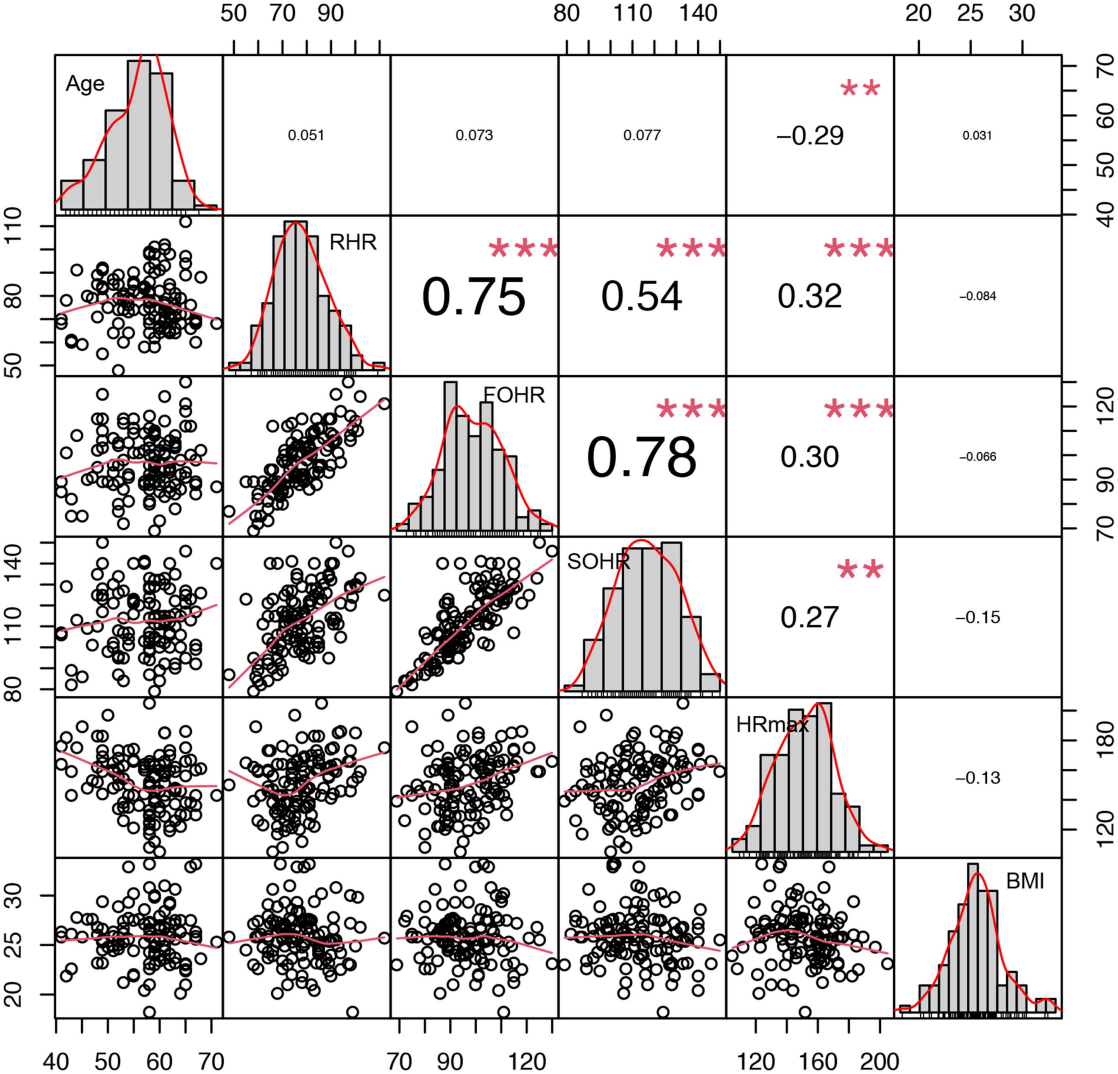
The correlation analysis between the maximal heart rate and other indicators. The scales in *x*-axis and *y*-axis are symmetric and indicate the values corresponding to indicators which align diagonally (units are dependent on indicators). The lower triangular areas display the distributions of indicator values by the scatter plots and fitted curves (solid red line), while the upper triangular areas show correlation coefficients between indicators, with asterisks representing the strengths of correlated relation.

HR_max_ was highly correlated with RHR and FOHR, but was least relevant with BMI (Figure 1). We also calculated the frequency distribution histograms of the RHR, first- and second-order heart rate, BMI, and age, respectively, which were aligned diagonally on the sub-panels in Figure 1. The scatter plots and fitted curves among the six indicators are shown in Figure 1.

Table 2 summarizes the bias between the estimated and observed values of the maximum heart rate obtained by the regression model and the ANN. First, a univariate linear regression was established as:


(1) $$HR_{\max}=201.77-0.88\times Age$$

**Table 2:** Regression models to predict HRmax.

Input variable	Accuracy	MSE
Age	10.31%	7.18
Age+SOHR+RHR	9.74%	5.71

RHR, resting heart rate; FOHR, first-order heart rate; and SOHR, second-order heart rate.

where the goodness-of-fit *R^2^ =0.083* and mean square error *MSE=7.18*. Meanwhile, a multivariate regression model, which incorporated the RHR, age, and SOHR, was rigorously presented as


(2) $$HR_{\max}=150.46+0.43\times RHR-0.96\times Age+0.20\times SOHR$$

with a goodness-of-fit *R^2^ =0.214* and mean square error *MSE=5.71*. Compared with the univariate regression model, predictions from the multivariate model were more precise because accuracy (defined as the ratio between the bias of observed and estimated values over the observed *HR_max_*) was 10.31 and 9.74%, respectively. The proposed regression model confirmed that the SOHR contributed to the maximum heart rate prediction. Nonetheless, multivariate regression was still unable to completely recapitulate the maximum heart rate from the three indicators because the goodness-of-fit was extremely low, which indicated that estimation of maximum heart rate was nonlinearly dependent on measurements.

## Discussion

In our study, an ANN approach was developed to explore nonlinearity. Four combinations were examined as input layer factors, representing the basic indicators of different individuals. Table 3 shows that the best accuracy reached 9.42%, which was significantly better than the regression models, and the MSE for the four combinations was significantly lower than those obtained from the univariate or multivariate models. Interestingly, instead of all indicators, the combination of age, RHR, and SOHR was the optimal variables during the process of ANN. It was heuristic that variable selection based on correlation analysis was critical to estimate heart rate *via* the neural network method.

**Table 3:** Artificial neural network (ANN) to predict HRmax.

Input variable	Accuracy	MSE
Age	9.85%	3.57
Age+FOHR+SOHR+RHR+BMI	9.57%	3.37
Age+RHR+BMI	9.56%	3.35
Age+SOHR+RHR	9.42%	3.23

RHR, resting heart rate; FOHR, first-order heart rate; and SOHR, second-order heart rate.

Both regression models and ANN methods were proposed to estimate the heart rate in this study. One similarity between them was using age as an independent variable, which is in line with research showing that age accounts for 35–80% of heart rate variation (Tanaka et al., 2001). In addition, both models confirm that the selected indicators (RHR, age, and SOHR) performed significantly better than single variables (Tables 2 and 3). The prediction accuracy of the neural network method was higher than that of regression models (Table 2) because of the self-learning and adaptive ability of the network property (Lins et al., 2017; Cognigni et al., 2018; Yang and He, 2018). The model parameters were updated during the minimization of the loss function through the forward propagation and back propagation phases. ANNs and other computational models are popular for predicting values in other sports science fields, such as player detection (Guo et al., 2020) and the investigation of exercise-mediated diseases (Tao et al., 2021), owing to their outstanding abilities for generalization and efficiency in investigating nonlinear latent relations between variables. During model establishment, four sets of variable combinations were verified based on the stepwise variable selection criteria (Figure 1). Our experiments also confirmed that there was no significant difference in the maximum heart rate between men and women (Table 1), which supports the conclusion of Tanaka et al. (2001) that no direct linkage between gender and the maximum heart rate was validated *via* meta-analysis after 351 studies (18, 712 participants included) were summarized. Meanwhile, BMI was less correlated with heart rate (Figure 1) in our experiments, which is consistent with the conclusions of Nes et al. (2013). However, since the sample size was relatively small in this study, it is not known whether BMI and gender are effective indicators to be included in the estimation model. Hence, this must be validated in future work.

## Data Availability Statement

The original contributions presented in the study are included in the article/supplementary material, further inquiries can be directed to the corresponding author.

## Ethics Statement

The studies involving human participants were reviewed and approved by the Ethics committee from Beijing Sport University. Written informed consent for participation was not required for this study in accordance with the national legislation and the institutional requirements.

## Author Contributions

KT, JhL, and WS designed the study. YL supervised the study. KT, JjL, WS, and HY performed the experiments. KT and JhL wrote the manuscript. All authors contributed to the article and approved the submitted version.

## Funding

This study was supported by grants from the National Key Research and Development Program of the Ministry of Science and Technology, China (2020YFC2002900, 2018YFC2000603), and the Natural Science Foundation of Beijing (grant number 1214023).

## Conflict of Interest

The authors declare that the research was conducted in the absence of any commercial or financial relationships that could be construed as a potential conflict of interest.

## Publisher’s Note

All claims expressed in this article are solely those of the authors and do not necessarily represent those of their affiliated organizations, or those of the publisher, the editors and the reviewers. Any product that may be evaluated in this article, or claim that may be made by its manufacturer, is not guaranteed or endorsed by the publisher.
